# A New Benchmark of Charges Storage in Single-Layer Organic Light-Emitting Diodes Based on Electrical and Optical Characteristics

**DOI:** 10.3390/molecules26030741

**Published:** 2021-01-31

**Authors:** Chengwen Zhang, Zheng Xu, Peng Wang, Zilun Qin, S. Wageh, Ahmed Al-Ghamdi, Suling Zhao

**Affiliations:** 1 Department of Physics, Faculty of Science, King Abdulaziz University, Jeddah 21589, Saudi Arabia; zhangchengwen6@126.com (C.Z.); AGAMDI@kau.edu.sa (A.A.-G.); 2 Key Laboratory of Luminescence and Optical Information, Ministry of Education, Beijing Jiaotong University, Beijing 100044, China; zhengxu@bjtu.edu.cn (Z.X.); 14121622@bjtu.edu.cn (P.W.); 14121620@bjtu.edu.cn (Z.Q.)

**Keywords:** organic light-emitting diodes, the storage of charges, transient electroluminescence

## Abstract

The storage of charges in organic light-emitting diodes (OLEDs) has drawn much attention for its damage to device performance as well as the loss to carriers. Thus, it is essential to address the issue and do further investigation. The traditional approach to storage analysis is mainly based on transient measurement since it is sensitive to transient instead of steady signal. In this paper, we proposed a new benchmark to investigate the single-layer OLEDs capable of stored charges with poly (methyl methacrylate) (PMMA), which is just based on electrical and optical characteristics. Since the stored charges contribute both to luminance and current of the devices with PMMA, the area between them can be taken as a benchmark and evaluated the storage of charges. In our experiment, the areas of 4 nm, 6 nm, 8 nm, and 10 nm PMMA devices are 0.348, 0.554, 0.808, and 0.894, respectively, indicating a higher capability of storage in thicker PMMA. It is exactly in line with the results taken from transient electroluminescence (EL) measurement. Thus, this new benchmark is practical and provides a more accessible approach to investigate the storage of charges in OLEDs.

## 1. Introduction

Organic light-emitting diodes (OLEDs) have developed rapidly in the recent several decades [1,2,3,4]. Especially in recent years, to achieve dramatic efficiency and excellent performance, great amounts of approaches have been adopted. For OLEDs, the balance of carrier transport is an imperative issue to address. Thus, more and more bipolar organic materials are under investigation like thermally activated delayed fluorescence (TADF) materials [5,6,7]. By manipulating the interplay between the wide-energy-gap host and the TADF sensitizer, the multiple sensitizing processes are triggered to eliminate the exciton losses with a high external quantum efficiency (EQE) of 24.2% [8]. Other approaches are put forward such as emitting dipole orientation (EDO) since it has an influence on the outcoupling efficiency of OLEDs [9,10]. The “hot exciton” mechanism, that is, hybridized local and charge-transfer (HLCT) has been brought out to balance the distribution between local excited (LE) state and charge-transfer (CT) state, which can promote more efficiency in blue OLEDs [11,12,13]. As to near-infrared OLEDs, to overcome the energy gap law and achieve high-performance, an exciton-vibration decoupling strategy has been proposed [14]. Additionally, newly emerged aggregation-induced delayed fluorescence (AIDF) has also advanced great performance. By drafting AIDF into common host materials, the efficient luminogens can harvest non-doped OLEDs both singlet and triplet excitons, and thus exhibit high-efficiency and negligible roll-off [15].

To further optimize device performance, advanced materials are not the only route, but to investigate the underlying physics of OLEDs is also of the essence. Among these processes, the carrier motion plays the leading role and is imperative to investigate. It is particularly worth noting the excess charges stored in bulk, which may originate from the charges’ accumulation near the interface [16] or the traps in localized states [17]. Moreover, they are ubiquitous and may even cause great damage to the device’s performance. First, excitons may be quenched by the extra polarons [18,19]. Second, it will lead to field-induced quenching because of the additional electric field created by these charges [20]. Third, the trap-assisted carriers will result in the nonradiative recombination to compete with the radiative one, and eventually, decrease the external quantum efficiency [21]. Finally, the storage of charges in the interface may lead to local overheating and even degradation [22]. Given these disadvantages, it is of vital importance to conduct further investigation on the mechanism of stored charges. Recently, more attention has been paid to this field.

Commonly, transient measurement is an optional approach to getting access to dynamic analysis on stored charges since excess charges will be sensitive to alternating signal. With respect to OLEDs, transient electroluminescence (EL) measurement is an appropriate way. When operating by pulse, the transient optical signal of OLEDs can reflect exciton emission, exciton quenching, and carrier capture or accumulation [19,23,24,25]. Especially for the EL spike, it can give more indication of stored charges [26,27,28,29]. By superposing tiny alternating stress to driving bias, Impedance spectroscopy (IS) or capacitance-voltage (C-V) characteristics can detect the response of excess charges and reveal the mechanism. Based on it, the defect states or carrier motion have been analyzed [30,31,32]. In addition, photo-capacitance has also attracted more attention [33,34,35]. Photo charge extraction by linearly increasing voltage (photo-CELIV) has been used to shed light on the ultra-long lifetime carrier stored in organic electronic devices [36,37]. A similar measurement based on metal-insulator-semiconductor CELIV (MIS-CELIV) is practical to measure the carrier mobility [38,39,40]. In this mode, carriers can be stored in the interface between insulator and semiconductor until the linearly increasing voltage is applied. Additionally, it is reported that light-induced electron spin resonance (ESR) spectroscopy can contribute to the analysis of charge accumulation states [41].

As can be seen, to investigate the storage of charges, various approaches have been carried out. All these measurements require high-accurate apparatus and elaborate design, which is practical but sort of sophisticated. In this paper, we proposed a new approach to investigate single-layer OLEDs capable of stored charges with PMMA, which is just based on current density-voltage-luminance (J-V-L) characteristics. It is indicated that the area between current and luminance is correlated to stored charges by PMMA. The transient EL measurement can also give solid evidence. Thus, this area can be taken as a benchmark to evaluate the storage in OLEDs, which is more accessible and enriches the approaches aimed at storage.

## 2. Results and Discussion

**Figure 1:**
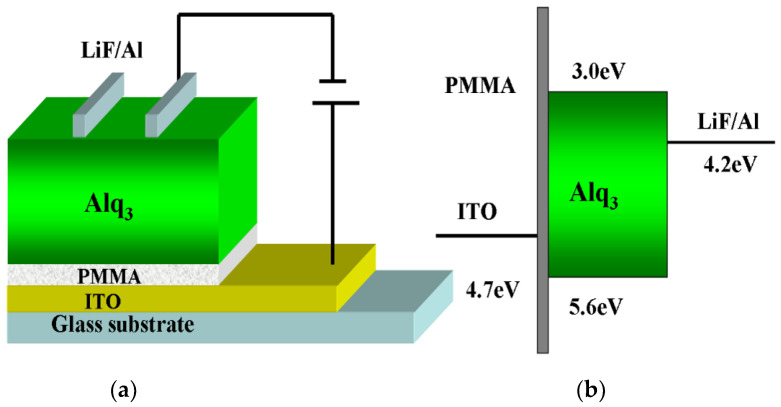
(**a**) Device structure of the OLEDs (**b**) Energy level diagram of the OLEDs [44].

To achieve storage, the selection of materials and device structure is worth noting. For organic materials, an unbalanced carrier is the leading cause of damage to the performance of the device. However, it also indicates a higher capability to form storage under specific conditions. Thus, it is feasible to choose a single-layer device. Herein, Tris-(8-hydroxyquinoline) aluminum (Alq_3_) is used as an emitting layer. Insulating materials with large bang gap and low conductivity can be used to confine the transport of carriers. In the memory device, the insulator poly (methyl methacrylate) (PMMA) has been reported as a good candidate for the capture of charges [42]. It can also be used in quantum-dot light-emitting diodes (QLEDs) to sustain the balance of carriers [43]. Thus, PMMA is picked to give rise to storage in Alq_3_-based OLEDs. As we know, the thickness of insulator film can dramatically impact the performance of devices. Accordingly, a series of PMMA films were prepared by spin coating with solutions of different concentrations at variable rotation rates. The film thickness was measured by an ellipsometer, which is exhibited in Table 1. In this paper, the parameters of approximate thicknesses of 4 nm, 6 nm, 8 nm, and 10 nm were taken as reference and the device structures of ITO (100 nm)/PMMA (4 nm, 6 nm, 8 nm, 10 nm)/Alq_3_(80 nm)/LiF (0.6 nm)/Al (80 nm) were prepared to give storage as mentioned in reference [44]. The PMMA-free device is the control device. The structure and energy level diagram of the OLEDs are shown in Figure 1a,b.

**Figure 2:**
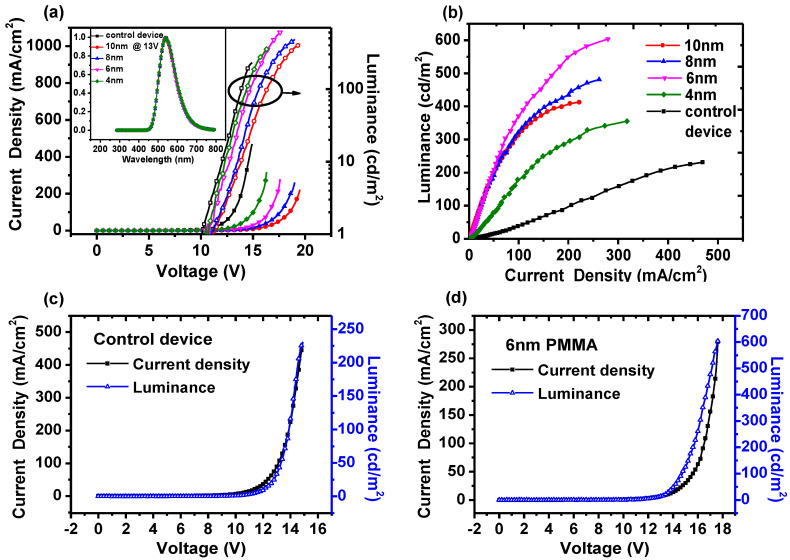
(**a**) J-V-L characteristics of all devices, inset is EL spectra of all devices operating under 13 V (**b**) L-J characteristics of all devices (**c**) J-V-L characteristics for control device (**d**) J-V-L characteristics for a device with 6 nm PMMA layer.

**Figure 3:**
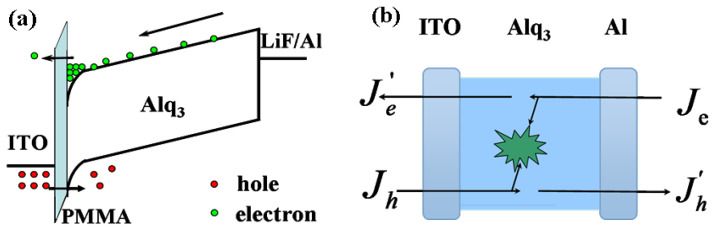
(**a**) The schematic diagram of influence of stored charges on carrier tunneling (**b**) the mechanism of current flowed in OLEDs.

The J-V-L characteristics and EL spectra of all devices are shown in Figure 2a. As can be seen, the EL spectra of all devices are unchanged and originated from Alq_3_ species when operating under 13 V, which indicates no influence of inserting a PMMA layer. Compared to the control device, the current density of the device with PMMA decreased remarkably. The more the PMMA thickness, the more its current density decreases, since PMMA can block off the transport of carriers due to its large band gap. However, devices with PMMA can achieve higher luminance than the control device with voltage increasing to high. The enhancement can be explained by the improved balance between holes and electrons. As for the control device, the Alq_3_-based device, its electron mobility is extremely high compared to its hole mobility [45,46]. Thus, it will eventually lead to incomplete recombination and large electron leak current. Whereas for devices with PMMA, on one side, redundant electrons will be blocked off by PMMA and provide sufficient electrons, on the other side, these charges will partly assist the injection of holes. Since all PMMA layers are ultrathin, the hole injection can behave as tunneling in high electrical field, which is shown in the schematic diagram of Figure 3a.

As the diagram shows, electrons stored by PMMA can form space charges and eventually influence the potential distribution as the Equation (1):

(1) $$V_{1}=\frac{(\varepsilon_{2}/d_{2})V+\sigma }{\varepsilon_{1}/d_{1}+\varepsilon_{2}/d_{2}},\;V_{2}=\frac{(\varepsilon_{1}/d_{1})V-\sigma }{\varepsilon_{1}/d_{1}+\varepsilon_{2}/d_{2}}$$

where *V* is the total applied voltage, *V*_1_ and *V*_2_ are the voltages dropped in PMMA and Alq_3_ respectively, *ε*_1_ and *ε*_2_ are the dielectric constants of PMMA and Alq_3_, *d*_1_ and *d*_2_ are the thicknesses of PMMA and Alq_3_, *σ* is the area density of the charges stored in the interface. The voltage drop in PMMA will increase for the existence of these stored charges, to some extent, which can facilitate hole injection for alignment of energy level.

Additionally, these stored charges can also change the local field nearby the interface. Its distribution shows the dependence on charge density according to Poisson’s equation

(2) $$\frac{dE}{dx}=\frac{q(p-n)}{\varepsilon \varepsilon_{0}}$$

where *E* is the electric field, *p* and *n* are the charge density of holes and electrons, respectively, *q* is the elementary charge, *ε*_0_ and *ε* are vacuum permittivity and dielectric constant of Alq_3_.

Given the large amount of stored electrons near the interface, the enhanced field induced by them will ultimately form the energy level bending as Figure 3a. Thus, it will enhance the hole injection for barrier weakening. To sum up, the charges stored by PMMA essentially give rise to brighter luminance than the control device.

Nevertheless, as can be seen, with PMMA increasing from 4 nm to 10 nm, the individual brightest luminance increased up to 6 nm and then gradually decrease, which can be accounted for the balance between hole injection and electron storage. The thin layer would contribute to the tunneling of holes but count against the blocking of redundant electrons. For the thick layer, it will behave in the opposite way. In either case, however, it cannot achieve sufficient recombination. Only in the case of the 6 nm PMMA can it meet the balance and thus achieve effective emitting.

Current originates from the motion of the carrier, and luminance is the direct reflection of photons. The dependence between luminance and current (L-J) can indicate the relative quantum efficiency with the slope of L-J [47], which is shown in Figure 2b. As can be seen, the slope of the device with PMMA is larger than that of the control device as expected. For 4 nm PMMA, the slope is lower than other PMMA devices, since it is of less capability to store electrons and thus can reap insufficient recombination and turn out low efficiency. It also indicates the dominance of charge storage in these devices. For 6 nm, 8 nm, and 10 nm PMMA, their slopes are almost identical in low current and then separate with current increasing. At a high current, the slope of the 6 nm PMMA device is larger. As the PMMA thickness increased, its slope gradually decreased, which may originate from hole tunneling since a higher electrical field can favor tunneling but the barrier width can also have a significant impact. It is worth noting that L-J for the control device is linear, however, such correlation for the PMMA device exhibits a curve profile. The thicker PMMA is, the more remarkable it is. To further investigate this phenomenon, the J-V-L characteristics of the control device and 6 nm PMMA are individually analyzed and shown in Figure 2c,d, respectively. The scales are adjusted to an appropriate extent for convenient analysis.

With respect to the control device, as can be seen, the profiles of current density and luminance are almost in line with each other except for the tiny discrepancy around 11 V. It can be illustrated by the mechanism of current flow in Figure 3b. Here *J_h_* and *J_e_* represent the injecting current density for holes and electrons. *J_h_’* and *J_e_’* represent the leak current density for holes and electrons. The total current density passed by the device can be shown as Equation (3):

(3) $$J=J_{h}+J_{e}{}^{\prime }=J_{e}+J_{h}{}^{\prime }$$

As the diagram shows, the difference between injected current and leak current is originated from recombination. Thus, the luminance can be expressed by Equation (4):

(4) $$L\propto J_{r}=J_{e}-J_{e}{}^{\prime }=J_{h}-J_{h}{}^{\prime }$$

As mentioned above, the electron mobility of Alq_3_ is dramatically higher than the hole mobility, thus the current of the control device can be express as *J_e_* + *J_h_’*. As for luminance, it can be assumed as the hole’s electric-field-induced recombination with the electron. Additionally, the amount of holes is less than the electrons, thus the recombination is dominated by holes and can be expressed as *J_h_* − *J_h_’*. At a low current, it can be found that *J_e_* and *J_h_* behave in an exponential pattern, but the carrier imbalance will generate insufficient recombination and large leak current *J_h_’*, which leads to the discrepancy of L-J. With injection increasing, the recombination is sort of improved so as to ease the leak current. Thus, *J_h_’* is negligible under a high field. Luminance and current can be expressed as *J_h_* and *J_e_*, which are approximately in line with each other as shown in Figure 2c. Evidently, it also indicates the linear correlation in Figure 2b.

With respect to the device with 6 nm PMMA, as can be seen in Figure 2d, obvious discrepancy between the luminance and the current taking place under a voltage higher than 13 V. In this region, the remarkable increase first took place in luminance under low current, and then, luminance and current both increased with gradually enhanced voltage. However, the former increased less rapidly than the latter and ultimately they both crossed. It thus evidently indicates the nonlinear correlation between luminance and current in Figure 2b. To investigate its mechanism, it is suggested to analyze current and luminance, respectively. As for current, the use of PMMA can lead to a large amount of electrons being stored by it. Accordingly, the current will decrease dramatically. With respect to luminance, on the contrary, this storage can ease the loss of the electrons so as to provide a sufficient electron reservoir. The recombination in organic semiconductors should follow the Langevin expression, whose recombination rate should be determined by the slowest charge carrier [48]. For this reason, the recombination of the Alq_3_-based control device is correlated to holes as we have discussed for the current. In the device with PMMA, however, these electrons stored by PMMA turned to be the slowest charge carriers and thus should dominate in the recombination. From a certain point of view, it can be regarded as electron-centered field-induced recombination. Therefore, a large amount of stored electrons will benefit from luminance. In addition, the injection of holes can also play a great role. As we mentioned above, the electrons stored by PMMA can partly assist the tunneling of holes in a high electrical field because of the rearrangement of energy levels. With all these combined efforts, it eventually gives rise to sufficient recombination. As a result, the different mechanisms between current and luminance can give rise to a discrepancy. It is worth noting that this discrepancy will diminish in higher voltage and lead to the cross between current and luminance as well as an enclosed region. It may originate from the exciton quenching induced by more stored charges [29], which can count against luminance. To sum up, the discrepancy of luminance and current is closely correlated to these stored charges. Thus, it is suggested to take the area between current and luminance profiles as a benchmark, so as to evaluate the storage of charges.

**Figure 4:**
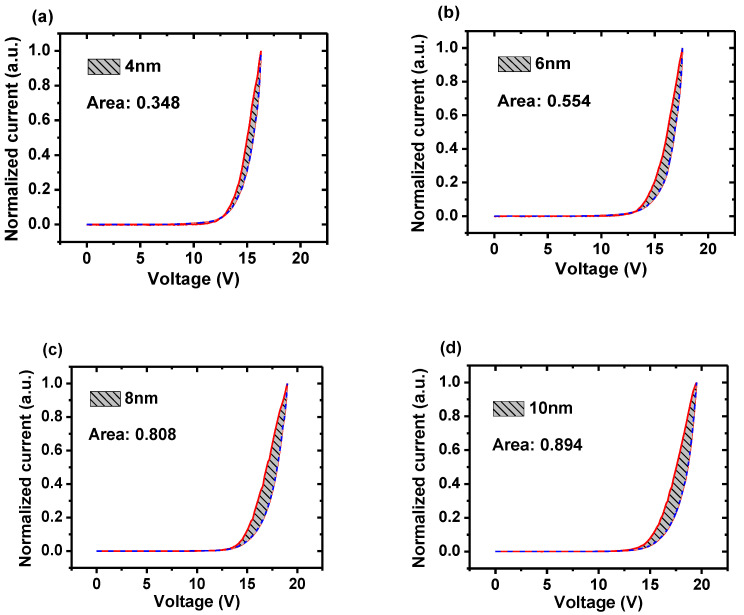
The normalized current and photo current of devices with PMMA of thicknesses of (**a**) 4 nm (**b**) 6 nm (**c**) 8 nm and (**d**) 10 nm. The areas between the two curves are 0.348, 0.554, 0.808, and 0.894, respectively.

**Figure 5:**
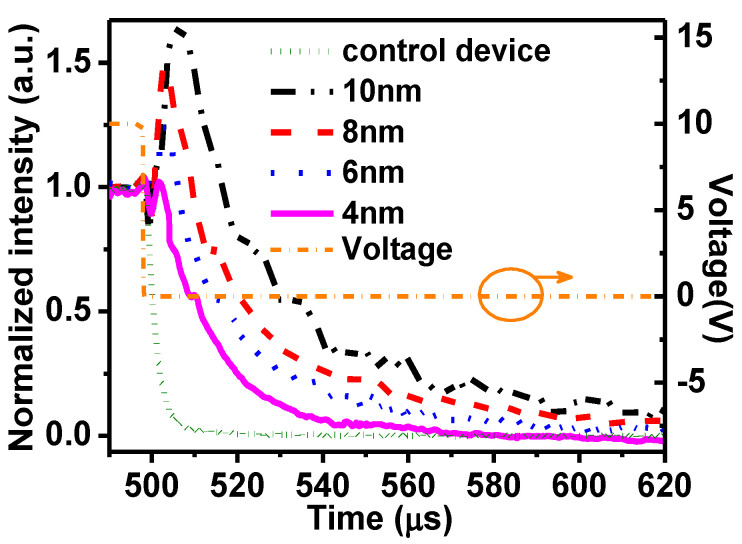
Transient EL measurements of all devices by applying a driving pulse of 10 V, 500 μs.

To analyze the storage of all devices with PMMA, the J-V-L characteristics are individually shown in Figure 4. In order to optimize the quantitative analysis, we convert luminance to photo current by the factor of spectral responsivity, which is 2 × 10^−4^ A/W in 530 nm for the Newport 1830 optical power meter. In addition, all characteristics are normalized. As can be seen, the device with 4 nm PMMA exhibits the smallest area of 0.348. As PMMA increases from 6 nm to 10 nm, the corresponding area is gradually enlarged and shown to be 0.554, 0.808, and 0.894. This indicates that the capability of charge storage will enhance with the increase in the thickness of PMMA. Since stored charges are sensitive to the alternating signal, transient EL measurement was carried out to give solid evidence. In an experiment, a series of transient pulses were generated by a waveform generator and applied to all the devices. The amplitude of the pulse was 10 V, which can last for 500 μs. All results were recorded by an oscilloscope, as shown in Figure 5.

For the control device, as can be seen, when the pulse ceased, its transient EL shows rapid decay immediately. For the device with PMMA, nevertheless, the EL intensity after the pulse was removed first decayed for an ultrashort time and then increased rapidly to give rise to an EL spike with a long decayed tail. Technically, based on our previous work, this should have originated from the storage of charges. When the device is operated under the driving pulse, the forward electrical field will drift electrons to the interface nearby PMMA, and then these electrons will be stored by PMMA. After the pulse is removed, the forward field will become extinct. However, these electrons cannot be stored instantaneously because of the existence of a built-in field. Thus, they were released in bulk. Meanwhile, the holes trapped in bulk were also set free from the located states so as to generate recombination and bring out the EL spike in Figure 5. For the device with PMMA, the results exhibit that the EL spike will be enhanced with the thickness of PMMA increasing, indicating the capability of storage for PMMA. It is exactly in line with the results of the area between the current and luminance mentioned above. Evidently, the evaluation we proposed can be taken as a benchmark of stored charges.

For the investigation of the stored charges, although the approach we proposed is similar to the transient measurement, there are still some distinctions. As for transient measurement, although it is practical, a fully functional system will utilize a large amount of apparatus in high-accuracy. Besides, the establishing of a transient system is somehow complicated. With respect to our research, it just relies on the basic measurement of current and luminance so as to evaluate the storage of charges, which is more feasible and accessible. However, it is still worth noting that our approach is only to estimate the capability of storage of a single-layer device. To investigate the charge motion, dynamic process, or storage in a multiple-layer device, it is suggested to turn to the help of functional transient measurement. Even so, the evaluation of storage in our work can be used as a benchmark to enrich the approaches aimed at storage and also provide a new perspective.

## 3. Materials and Methods

The devices were fabricated on patterned indium tin oxide (ITO) with a sheet resistance 20 Ω/□. The structure was carried out as ITO/poly (methyl methacrylate) (PMMA) (4 nm, 6 nm, 8 nm, 10 nm)/Tris-(8-hydroxyquinoline) aluminum (Alq_3_) (80 nm)/LiF (0.6 nm)/Al (80 nm), and the control device was PMMA-free. The ITO substrate was cleaned with detergent water, ethanol, and de-ionized water in sequence by an ultrasonic cleaner and dried with nitrogen, then followed by a UV-ozone treatment for 10 min. PMMA was dissolved in chloroform to prepare the solution with a concentration of 1 mg/mL and 0.5 mg/mL. Then both solutions were spin-coated onto ITO at the rotation rate from 1000 rpm to 6000 rpm. The thickness of PMMA film is measured by an ellipsometer in a silicon substrate. Alq_3_ was thermally evaporated at a pressure of 5 × 10^−4^ Pa. Then, LiF and Al were sequentially deposited under a high vacuum condition of 2 × 10^−4^ Pa through a shadow mask. Their thicknesses were monitored and controlled with quartz crystal monitors. The active area of the device is 0.09 cm^2^.

The current density-voltage-luminance (J-V-L) characteristics were measured with a programmable Keithley Source Meter 2410 (Beaverton, OR, USA) and Newport 1830 Optical Power Meter (Irvine, CA, USA). The electroluminescence (EL) spectrum was detected by a charge-coupled device (CCD) spectrometer. The transient EL was performed under forward pulses, which were generated by RIGOL DG1022 Function/Arbitrary Waveform Generator (Beijing, China). The characteristics of transient EL were detected by the Zolix Instruments Model PMTH-S1C1-CR131 Photomultiplier Tube (Beijing, China) and recorded with the Tektronix Model DPO 4104 digital phosphor oscilloscope (Beaverton, OR, USA). All measurements were carried out at room temperature under ambient atmosphere.

## 4. Conclusions

In summary, a new benchmark of charge storage based on electrical and optical characteristics was proposed for single-layer OLEDs with PMMA. Unlike the linear correlation in the control device, the discrepancy took place between the current and luminance of devices with PMMA. The investigation indicates that the stored charges play a great role in both current and luminance. Thus, it is proposed that the area between luminance and current can be taken as a benchmark to evaluate the storage of charges. Based on the analysis, it was found that this area will enlarge with the increase in PMMA, indicating a higher capability of stored charges to thicker PMMA. This is exactly in line with the results taken from transient EL measurement. Thus, this new benchmark is practical and accessible to investigate the storage of charges in OLEDs.

## Figures and Tables

**Table 1:** The thickness of spin-coated PMMA film measured by an ellipsometer.

	Rotation Rate (rpm)	1000	3000	5000	6000
Concentration (mg/mL)					
1		102.89 Å	87.19 Å	82.38 Å	×
0.5		62.66 Å	45.54 Å	×	39.93 Å

×: films were not fabricated under such conditions.

## Data Availability

The data presented in this study are available in manuscript.

## Sample Availability

Samples of the compounds are not available from the authors.
